# A 3D analytical ion transport model for ionic polymer metal composite actuators in large bending deformations

**DOI:** 10.1038/s41598-021-85776-4

**Published:** 2021-03-19

**Authors:** Mohsen Annabestani, Nadia Naghavi, Mohammad Maymandi-Nejad

**Affiliations:** Department of Electrical Engineering, Ferdowsi University of Mashhad, Mashhad, Iran

**Keywords:** Engineering, Mathematics and computing

## Abstract

Ionic polymer metal composites (IPMCs) are a kind of soft electroactive polymer composites. An IPMC strip commonly has a thin polymer membrane coated with a noble metal as electrodes on both sides. Whenever an electric voltage is applied to the IPMC, it bends and whenever it is deformed, a low voltage is measurable between its electrodes, hence IPMC is an actuator as well as a sensor. They are well known for their promising features like low density, lightness, high toughness and remarkable stimulus strain, also, they have the potential for low-voltage operation while exhibiting acceptable large bending deformation. In this paper, a three-dimensional (3D), dynamic and physics-based model is presented analytically and experimentally for IPMC actuators. The model combines the ion transport dynamics within the IPMC and the bending dynamics of it as a beam under an electrical stimulation. In particular, we present an analytical model to create a relation between the input voltage and the output tip displacement of an IPMC actuator for large bending deformations. Experimental results show that the proposed model captures well the tip displacement.

## Introduction

**Figure 1:**
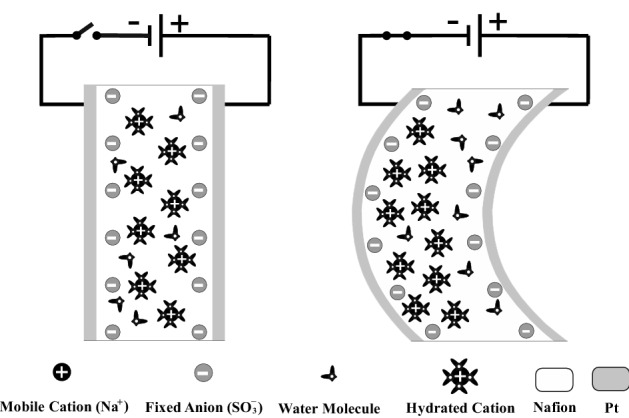
Working principle of an IPMC actuator: (Left) before applying an electrical field; (Right) after applying an electrical field.

Ionic polymer metal composites (IPMCs) are one of the main ionic electro-active polymers that have potential applications especially in the field of medicine^1–6^. As depicted in Fig. 1, an IPMC strip has a thin polymer membrane (e.g. Nafion) coated with a noble metal as electrodes (e.g. Pt) on both sides (anode and cathode). A relatively low voltage applied through the thickness of an IPMC causes the internal hydrated cations to transport toward the cathode. This transportation leads to the Nafion inflation in the cathode side and consequently produces a bending response toward the anode side^7–11^. Reversely, we can measure a low voltage signal between its electrodes in response to an input mechanical deformation, hence IPMC is an actuator and a sensor as well. IPMCs have received great interest in recent years for their potential applications both as sensor and actuator in a large variety of engineering areas. They are bio-compatible^5^ and require low voltages (less than 5 V), produce large bending deformations, can work properly in air and in aqueous environments using appropriate encapsulation layers^6^, and have very large stimulus strain and low density^1^. As mentioned in the advantages of an IPMC actuator, it can produce large bending deformations, however, in this situation, its behavior is nonlinear^12–15^. IPMC’s behavior is time-dependent and except its steady state analysis, it should be modeled dynamically which means that static models cannot be used for tracking the time-dependent performance of an IPMC. Additionally, IPMC shows nonlinear behavior in large deformation situations. Hence, it is required to use dynamic nonlinear models to reach a time-dependent model in large deformation situations of IPMC. In recent years, there have been extensive efforts in the dynamic modeling and understanding of IPMC actuators, however, they have studied small bending deformations (tip displacement of less than 1 mm) in their experiments and have not discussed large bending deformations. Although, due to the widespread use of IPMC actuators, an accurate and practical dynamic model is desirable. In general, approaches for the modeling of IPMC actuators can be divided into two main groups. The first group includes analytical modeling and the second one consists of predictive identification methods. Various analytical models have been studied for IPMC actuators. For example, distributed resistor–capacitor (RC) equivalent circuits^15–18^, physical and multi-physical approaches using partial differential equations (PDEs), finite element methods, COMSOL modeling^19–21^ and also several miscellaneous uncategorized models^22,23^ are some of the presented analytical models. Moreover, there are two approaches in the predictive identification group, classical^24,25^ and intelligent methods^9,10,26^. Nevertheless, the important point is that all mentioned methods in the two main groups have not addressed the issue of large bending deformations of an IPMC actuator except^12^. On the other hand, reference^12^ has proposed a different predictive identification method but this method is completely a black-box model and cannot describe the dynamic chemo-electric response of an IPMC actuator.

The contribution of the current paper is an analytical, three-dimensional (3D), dynamic and physics-based model which can describe the true electromechanical behavior of IPMC actuators even in large bending deformations. Various physics-based models have also been studied for IPMCs which can be classified into three categories, thermodynamics of irreversible process (TIP) models^25–27^, frictional models (FR)^20,21^ and Nernst-Planck based (NP) models^28–33^. Among these three categories, NP models propose the most straightforward way to explain ion transport in IPMC sensors and actuators.

In the current paper, the starting point of the model development is the same governing PDE as in NP-based models presented until now. However, this work improves previous studies significantly in three aspects. First, it considers that the ionic content of an IPMC strip migrates through all directions and needs to solve the problem in a three-dimensional space. All the above mentioned NP-based methods are one dimensional except^28^ which is a two dimensional, non-analytical and semi-numerical method. Second, the proposed model is the first dynamic and full analytical 3D physics-based ion transport model that covers the nonlinear behavior of IPMC actuators even in large bending deformations. Third, it is a promising result that for the first time we get a mathematical well-defined relationship between the input voltages applied to the IPMC and the curvature of the IPMC in large bending deformations.

Experiments were conducted to validate the proposed dynamic model for an IPMC actuator in a cantilever configuration. A good match was achieved between the measured bending response and the model prediction.

The rest of this paper consists of three main sections. In Section II we describe our proposed 3D physics-based ion transport model and its details. The estimation of the model’s unknown parameters and assessment of the model accuracy are presented in Section III. The discussion along with a conclusion is provided in Section IV.

## A 3D physics-based ion transport model for IPMC

### Preliminary equations and obtaining the governing partial differential equations

**Figure 2:**
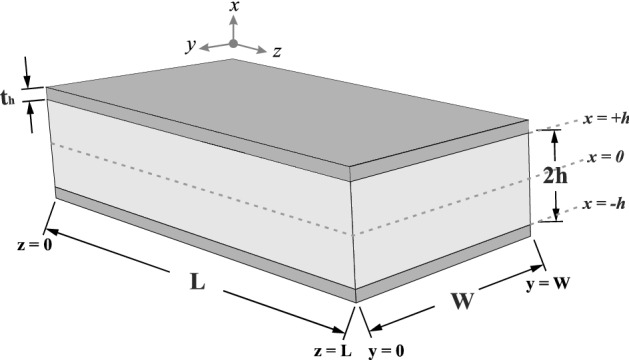
The geometric definition of an IPMC beam.

A geometric definition of an IPMC beam is illustrated in Fig. 2. The beam is clamped at one end (z = 0) and is subject to an electrical voltage. In the following equations, the electric displacement, the electric field, the electric potential and the electric charge density are denoted, respectively, by ${\mathbf{D}}\left( {x,y,z,t} \right),\,\,{\mathbf{E}}(x,y,z,t),\,\,\phi (x,y,z,t)\,$ and $\rho \left( {x,y,z,t} \right)$. The following equations hold:

1 $${\mathbf{E}}\left( {x,y,z,t} \right) = \frac{{{\mathbf{D}}\left( {x,y,z,t} \right)}}{{\kappa_{e} }}\,\,,$$

2 $${\mathbf{E}}\left( {x,y,z,t} \right) = - \nabla \phi \left( {x,y,z,t} \right)\,\,,$$

3 $$\nabla .\,{\mathbf{D}}\left( {x,y,z,t} \right) = \rho \left( {x,y,z,t} \right)\,\,,$$

where $\left( {x,y,z} \right) \in \,\,\,\left[ { - h, + h} \right]\,\, \times \,\,\,\left[ {0,W} \right]\,\,\, \times \,\,\,\left[ {0,L} \right]$, $\kappa_{e}$ is the effective dielectric constant of the membrane and $\rho \left( {x,y,z,t} \right)$ is also defined as follows ^29–31^:

4 $$\rho \left( {x,y,z,t} \right) = F\left( {C^{ + } \left( {x,y,z,t} \right) - C^{ - } } \right).$$

In (4), *F* is the Faraday constant and $C^{ + } \left( {x,y,z,t} \right)$ and $C^{ - }$ are cation and anion concentrations in the membrane, respectively.

The continuity equation for cations is given by:

5 $$\nabla \cdot {\mathbf{J}}\left( {x,y,z,t} \right) = - \frac{{\partial C^{ + } \left( {x,y,z,t} \right)}}{\partial t}\,\,,$$

that ${\mathbf{J}}\left( {x,y,z,t} \right)$ is the cation flux vector. The ion flux consists of diffusion, migration and convection terms in the Nernst-Planck PDE as follows:

6 $${\mathbf{J}} = - d\left( {\nabla C^{ + } + \frac{{C^{ + } F}}{RT}\nabla \phi + \frac{{C^{ + } \Delta V}}{RT}\nabla p} \right) + C^{ + } {\mathbf{v}}\,\,,$$

where *d* is the ionic diffusivity, *R* is the gas constant, *T* is the absolute temperature, *p* is the fluid pressure, **v** is the free solvent velocity field and $\Delta V$ represents the volumetric change of the membrane^31,32^.

*Note* As considered until now, in this model all variables are assumed three-dimensional time-variant. However, for ease of notation, (x, y, z, t) is omitted in the following equations, except in equations that may lead to confusion.

Equation (7) is a time-variant 3D PDE that describes the electric potential of IPMC,$\phi \left( {x,y,z,t} \right)$. For more details, we refer the reader to Appendix A.

7 $$\frac{\partial }{\partial t}\left( {\nabla^{2} \phi } \right) = d\,\nabla^{2} \left( {\nabla^{2} \phi } \right) - K\left( {\nabla^{2} \phi } \right)$$

The objective of the next part is to solve Eq. (7).

#### Lemma 1

*Equation (**7**) is a generalized expression of the PDE that Nemat-Nasser obtained for *$\phi \left( {x,t} \right)$*, for the first time in 2000*^31^*. Extending Eq. *(7) *just for x variable, results in Eq.* (8)* which is exactly the same as the PDE that Nemat-Nasser got for *$\phi \left( {x,t} \right)$*(we refer the reader to Eq. 37 in*^31^*).*

8 $$\frac{{\partial^{3} \phi }}{{\partial x^{2} \partial t}} = d\,\left( {\frac{{\partial^{4} \phi }}{{\partial x^{4} }} - a^{2} \frac{{\partial^{2} \phi }}{{\partial x^{2} }}} \right)$$

9 $$a = \sqrt {\frac{{F^{2} C^{ - } \left( {1 - C^{ - } \Delta V} \right)}}{{RT\kappa_{e} }}} \,\,.$$

### Finding a solution for $\phi \left( {x,y,z,t} \right)$

The solution of Eq. (7),$\phi \left( {x,y,z,t} \right)$, represents the electric potential for the IPMC. It is obvious that the electric potential difference between the surface electrodes at the clamped end of the IPMC is similar to the applied input voltage, $V_{I} \left( t \right)$. Thus, the governing boundary conditions for this boundary problem are expressed as follows.

10 $$\left\{ \begin{gathered} \phi \left( { + h,0,0,t} \right) = \frac{{V_{I} \left( t \right)}}{2} \hfill \\ \phi \left( { - h,0,0,t} \right) = - \frac{{V_{I} \left( t \right)}}{2} \hfill \\ \phi \left( { + h,0,L,t} \right) = a_{L} \left( {V_{I} \left( t \right)} \right)\frac{{V_{I} \left( t \right)}}{2} \hfill \\ \phi \left( { - h,0,L,t} \right) = - a_{L} \left( {V_{I} \left( t \right)} \right)\frac{{V_{I} \left( t \right)}}{2} \hfill \\ \end{gathered} \right.\,\,\,,\,\,\,\left\{ \begin{gathered} \phi \left( { + h,W,0,t} \right) = \frac{{V_{I} \left( t \right)}}{2} \hfill \\ \phi \left( { - h,W,0,t} \right) = - \frac{{V_{I} \left( t \right)}}{2} \hfill \\ \phi \left( { + h,W,L,t} \right) = a_{L} \left( {V_{I} \left( t \right)} \right)\frac{{V_{I} \left( t \right)}}{2} \hfill \\ \phi \left( { - h,W,L,t} \right) = - a_{L} \left( {V_{I} \left( t \right)} \right)\frac{{V_{I} \left( t \right)}}{2} \hfill \\ \end{gathered} \right.$$

where the function $a_{L} \left( {V_{I} \left( t \right)} \right)$ is called distributed surface attenuation. Ideally, if IPMC electrodes are assumed perfectly conducting surface and the fractal penetration of the electrodes into the membrane is ignored, the voltage of the clamped region will be constant along the length of the electrodes without any attenuation in its longitudinal direction ^23^. Since IPMC electrodes are not perfectly conducting surfaces due to the electrode deposition process and also fractal penetration of the electrodes into the membrane, the applied voltage is attenuated along the *z*-axis (Fig. 2) proportional to the amplitude, frequency and other components of the input voltage.$a_{L} \left( {V_{I} \left( t \right)} \right)$ is a continuous function that produces a real number in the $\left( {0,1} \right]$ interval and $a_{L} \left( {V_{I} \left( t \right)} \right) = 1$ means electrodes are perfectly conducting surfaces.

To obtain an analytical solution for Eq. (7), we start from an assumption that the answer is a separable function which leads to Eq. (11). For more details that support the achievements presented in Eqs. (11–19), we refer the reader to Appendices B and C.

11 $$\,\phi \left( {x,y,z,t} \right) = k_{1} e^{ - \lambda t} \left[ {U_{1} \left( {x,y,z,t} \right) + \,U_{2} \left( {x,y,z} \right)} \right]$$

That $U_{1} \left( {x,y,z,t} \right)$ and $U_{2} \left( {x,y,z} \right)$ are defined as Eq. (12) and Eq. (13), respectively.

12 $$\begin{aligned} \,\,\,U_{1} \left( {x,y,z,t} \right) &= \left\{ {\frac{{V_{I} \left( t \right)}}{2}\left( {\frac{{Sinh\left( {\hat{\alpha }_{1} \left( t \right)x} \right)}}{{Sinh\left( {\hat{\alpha }_{1} \left( t \right)h} \right)}}} \right)} \right. \hfill \\ &\quad \times \left( {\left( {\frac{{1 - \cosh \left( {\hat{\alpha }_{2} W} \right)}}{{Sinh\left( {\hat{\alpha }_{2} W} \right)}}} \right)Sinh\left( {\hat{\alpha }_{2} y} \right) + \cosh \left( {\hat{\alpha }_{2} y} \right)} \right) \hfill \\ &\quad \times \left( {\frac{{a_{L} \left( {V_{I} \left( t \right)} \right) - cosh\left( {\hat{\alpha }_{3} \left( t \right)L} \right)}}{{sinh\left( {\hat{\alpha }_{3} \left( t \right)L} \right)}}} \right) \hfill \\ &\quad\left. { \times \left( {sinh\left( {\hat{\alpha }_{3} \left( t \right)z} \right) + cosh\left( {\hat{\alpha }_{3} \left( t \right)z} \right)} \right)\,} \right\} \hfill \\ \end{aligned}$$

13 $$\begin{gathered} \,\,\,\,\,\,\,\,\,\,\,\,\,\,\,\,\,\,\,\,U_{2} \left( {x,y,z} \right) = \sum\limits_{m = 0}^{\infty } {\sum\limits_{n = 0}^{\infty } {\sum\limits_{p = 0}^{\infty } {a_{mnp} \,\Xi \left( {x,y,z} \right)\,\,} } } \hfill \\ \mathrel\backepsilon \,\,\,\,\Xi \left( {x,y,z} \right) = \sin \frac{{\left( {m + 1} \right)\pi }}{2h}x\,\,\sin \frac{{\left( {n + 1} \right)\pi }}{W}y\,\,\sin \frac{{\left( {p + 1} \right)\pi }}{L}z \hfill \\ \end{gathered}$$

$k_{1}$ and $\lambda$ are constant coefficients and $\hat{\alpha }_{1} \left( t \right)$,$\hat{\alpha }_{2}$ and $\hat{\alpha }_{3} \left( t \right)$ are defined as follows:

14 $$\hat{\alpha }_{1} \left( t \right) = \sqrt {\hat{K} - \left\{ {\frac{{\mu^{2} }}{{W^{2} }}\left( {Ln\left( {1 - \frac{1}{{\mu^{2} }}} \right)} \right)^{2} + \frac{2\theta }{L}Ln\left( {\frac{4}{{a_{L} \left( {V_{I} \left( t \right)} \right)L\theta }}} \right)} \right\}} \,,$$

15 $$\hat{\alpha }_{2} = \frac{\mu }{W}Ln\left( {1 - \frac{1}{{\mu^{2} }}} \right)\,\,,$$

16 $$\hat{\alpha }_{3} \left( t \right) = \sqrt {\frac{2\theta }{L}Ln\left( {\frac{4}{{a_{L} \left( {V_{I} \left( t \right)} \right)L\theta }}} \right)} \,\,,$$

In the above equations, $\theta$ is $\frac{1}{W}$ and $\mu$ is a constant coefficient that should be bigger than 1. Also $\hat{\alpha }_{1} \left( t \right)$,$\hat{\alpha }_{2}$ and $\hat{\alpha }_{3} \left( t \right)$ have to always satisfy the following condition.

17 $$\hat{\alpha }_{1} \left( t \right)^{2} + \hat{\alpha }_{2}^{2} + \hat{\alpha }_{3} \left( t \right)^{2} = \hat{K}$$

In Eq. (13), $a_{mnp}$ is defined by Eq. (18).

18 $$a_{mnp} = \,\frac{{\frac{{8\hat{K}}}{2hWL}\int_{ - h}^{ + h} {\int_{0}^{W} {\int_{0}^{L} {\tilde{\Psi }} } } \left( {x,y,z} \right)\,dx\,dy\,dz}}{{\left( {\left( {\frac{{\left( {m + 1} \right)\pi }}{2h}} \right)^{2} + \left( {\frac{{\left( {n + 1} \right)\pi }}{W}} \right)^{2} + \left( {\frac{{\left( {p + 1} \right)\pi }}{L}} \right)^{2} + \hat{K}} \right)}}$$

where

19 $$\begin{gathered} \tilde{\Psi }\left( {x,y,z} \right) = \Psi \left( {x,y,z} \right)\, \hfill \\ \,\,\,\,\,\,\,\,\,\,\,\,\,\,\,\,\,\,\,\,\,\,\,\,\, \times \left( {\sin \frac{{\left( {m + 1} \right)\pi }}{2h}x\,\,\sin \frac{{\left( {n + 1} \right)\pi }}{W}y\,\,\sin \frac{{\left( {p + 1} \right)\pi }}{L}z} \right)\,, \hfill \\ \end{gathered}$$

and

20 $$\begin{gathered} \Psi \left( {x,y,z} \right) = \Upsilon_{1} \,x + \Upsilon_{2} \,y + \Upsilon_{3} \,z + \Upsilon_{4} \,xy + \Upsilon_{5} \,xz \hfill \\ \,\,\,\,\,\,\,\,\,\,\,\,\,\,\,\,\,\,\,\,\,\, + \Upsilon_{6} \,yz + \Upsilon_{7} \,xyz + \Upsilon_{8} \, + \frac{{\gamma_{1} }}{2}x^{2} + \frac{{\gamma_{2} }}{2}y^{2} + \frac{{\gamma_{3} }}{2}xz^{2} \,. \hfill \\ \end{gathered}$$

$\Upsilon_{1}$ to $\Upsilon_{8}$ and $\gamma_{1}$ to $\gamma_{3}$ are constant coefficients.

#### Lemma 2

*By extending Eq.* (11) *just for a small amount of x (represents a small bending deformation for the IPMC) and by ignoring y and z, it changes to the following equation which is the exact equation that Farinholt*^30,33^* found for the electric potential.*

21 $$\phi \left( {x,t} \right) = \frac{{\rho_{0} }}{{\kappa_{e} \hat{\alpha }_{1}^{2} \sinh \left( {\hat{\alpha }_{1} h} \right)}}\left( {\sinh \left( {\hat{\alpha }_{1} x} \right) - \frac{x}{h}\,\sinh \left( {\hat{\alpha }_{1} h} \right)} \right)e^{ - \lambda t}$$

$\rho_{0}$ is the boundary value of $\rho \left( {x,t} \right)$ in *x* = *h*. Indeed, Eq. (20) that supports large bending deformations is a generalized form of the equation presented by Farinholt (Eq. 21) for small bending deformations.

### The electro-mechano-chemical coupling

As mentioned in Section I, when a voltage is applied to the IPMC, hydrated sodium cations move to the cathode side in response to the applied input voltage across the membrane. This migration leads to increasing concentration of water molecules in the cathode side and gradually decreases them in the anode side. When the cathode negative charge equals the positive charge of sodium ions, this motion is stopped. Increasing water molecules in the cathode side leads to the Nafion inflation around the cathode and consequently, actuation toward the anode is observed^23,33^. Hence, to create an electro-mechano-chemical coupling, the first step is to calculate the concentration of cations (electrochemical component) and then the next step is to find a charge–stress coupling (electromechanical component). Nemat-Naser and Li^31^ assumed that the charge density at the surface of the polymer (*ρ*) is proportional to the induced stress (*σ*) in the form of *σ*(*x,t*) = *αρ*(*x,t*)*,* where *α* is the charge–stress coupling constant. This assumption was utilized in the most papers^16,29,30,32,33^ in the field of IPMC modeling. Therefore, one obtains the following equation at the surface of the polymer (*x* =  ± *h*):

22 $$\begin{aligned} \rho \left( {x,t} \right) & = FC^{ + } \left( {x,t} \right) - FC^{ - } \mathop{\longrightarrow}\limits^{{\sigma \left( {x,t} \right) = \alpha \rho \left( {x,t} \right)}} \\ & \quad \to \sigma \left( {x,t} \right) = \left( {\alpha F} \right)C^{ + } \left( {x,t} \right) - \left( {\alpha FC^{ - } } \right)\,. \\ \end{aligned}$$

However, we consider a three-dimensional time-variant form of this assumption, which will be shown to produce more accurate predictions in experiments, especially for large bending deformations.

**Figure 3:**
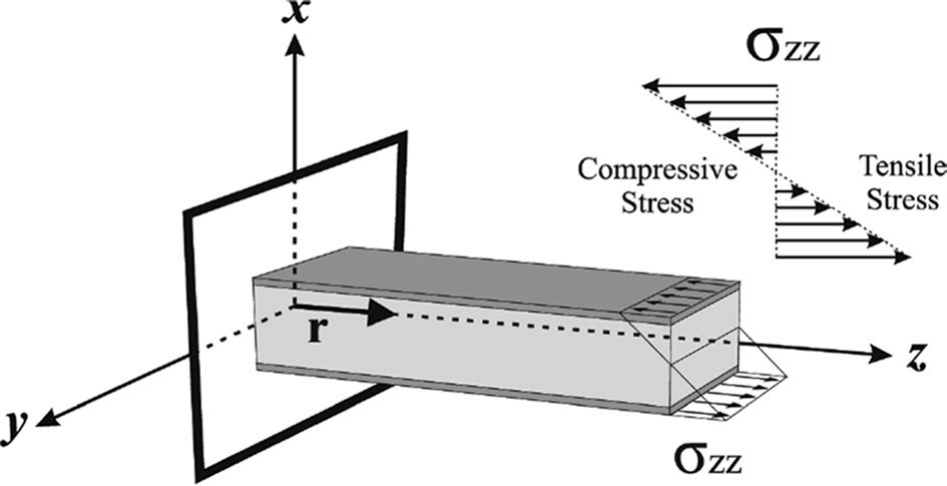
To cause bending in the direction of the *x*-axis (the main direction of the IPMC’s bending), mechanical stress should be induced in the direction of the *z*-axis.

As shown in Fig. 3, based on the governing principles in mechanics for a cantilever beam (i.e. an IPMC), bending in the direction of the *x*-axis (the main direction of the IPMC bending) needs a component of an induced stress in the direction of the *z*-axis. On the other hand, the most of papers which presented a physical model for IPMC actuators assumed that cations move in the direction of the *x*-axis between the anode and cathode. It means they assumed that the concentration of cations is a one-dimensional variable^16,19,35–41^. Nevertheless, in this paper, we modified this assumption and extended it to a three-dimensional time-variant problem. Hence, it is assumed that ions move in the direction of vector **A**. Components of vector **A** depend on the electric resistance of the IPMC, which is defined as follows:

23 $${\mathbf{A}} = \Omega_{x} \left( {r_{M} ,t} \right){\mathbf{i}} + \Omega_{y} \left( {r_{ew} ,t} \right){\mathbf{j}} + \Omega_{z} \left( {r_{el} ,t} \right){\mathbf{k}}\,.$$

In (23), $\Omega_{x} \left( {r_{M} ,t} \right)$, $\Omega_{y} \left( {r_{ew} ,t} \right)$ and $\Omega_{z} \left( {r_{el} ,t} \right)$ are functions of the membrane resistance (*r*_*M*_), the transverse resistance (*r*_*ew*_) and the longitudinal resistance (*r*_*el*_) of electrodes, respectively.

Indeed, migration and concentration changes of cations, which lead to mechanical stress and bending of the IPMC, are done in the direction of vector **A**. Mathematically, to calculate the changes of a multivariable function in the direction of a vector, the directional derivative of the function should be calculated in the direction of the desired vector. For example, to find the changes of ion’s concentration in the direction of the movement’s vector, the directional derivative of $C^{ + } \left( {x,y,z,t} \right)$ in the direction of vector **A** should be calculated. It is shown by $\nabla_{{\mathbf{A}}} C^{ + } \left( {x,y,z,t} \right)$ and defines as follows:

24 $$\nabla_{{\mathbf{A}}} C^{ + } = \frac{{\partial C^{ + } }}{{\partial {\mathbf{A}}}} = {\mathbf{a}}.\nabla C^{ + } \,,$$

**a** is the unit vector of **A** and defined by ${\mathbf{a}} = \frac{{\mathbf{A}}}{{\left| {\mathbf{A}} \right|}}$. Also,$\nabla C^{ + }$ is a gradient of $C^{ + } \left( {x,y,z,t} \right)$.

Now, if it is considered that the bending displacement of a stimulated IPMC is attenuated over the time due to the resistance, hence it is sensible to choose time-ascending functions for modeling effects of the resistance (functions like a ramp, sigmoid, tangent hyperbolic, etc.). In the following, for Eqs. (25–27), we choose a ramp function for $\Omega_{x} \left( {r_{M} ,t} \right)$, $\Omega_{y} \left( {r_{ew} ,t} \right)$ and $\Omega_{z} \left( {r_{el} ,t} \right)$ and we define the mentioned resistances as functions of dimensions (*h*, *W*, and *L*).

25 $$\Omega_{x} \left( {r_{M} \left( h \right),t} \right) = r_{M} \left( h \right)\,t\,u(t)$$

26 $$\Omega_{y} \left( {r_{ew} \left( W \right),t} \right) = r_{ew} \left( W \right)\,t\,u(t)$$

27 $$\Omega_{z} \left( {r_{el} \left( L \right),t} \right) = r_{el} \left( L \right)\,t\,u(t)$$

where *u*(*t*) is the Heaviside step function. Consequently, $\nabla_{{\mathbf{A}}} C^{ + }$ is obtained as follows:

28 $$\nabla_{{\mathbf{A}}} C^{ + } = \frac{1}{{\hat{r}\left( {h,W,L} \right)}}\left( {r_{M} \left( h \right)\frac{{\partial C^{ + } }}{\partial x} + r_{ew} \left( W \right)\frac{{\partial C^{ + } }}{\partial y} + r_{el} \left( L \right)\frac{{\partial C^{ + } }}{\partial z}} \right),$$

and $$\hat{r}\left( {h,W,L} \right)$$ is:

29 $$\hat{r}\left( {h,W,L} \right) = \sqrt {r_{M} \left( h \right)^{2} + r_{ew} \left( W \right)^{2} + r_{el} \left( L \right)^{2} } \,.$$

$\nabla_{{\mathbf{A}}} C^{ + }$ determines changes in the cations concentration in the direction of the optimum movement’s vector. These changes induce the mechanical stress. Hence, there is a linear relationship between $\nabla_{{\mathbf{A}}} C^{ + }$ and the mechanical stress:

30 $$\sigma \left( {x,y,z,t} \right) = \sigma_{0} \,\nabla_{{\mathbf{A}}} C^{ + } \left( {x,y,z,t} \right)\,.$$

That $\sigma_{0}$ is a constant coupling coefficient that should be estimated in the model estimation stage.

### The mechanical stress

To find the induced stress using Eq. (30) we should calculate $\nabla_{{\mathbf{A}}} C^{ + }$ and for calculation of $\nabla_{{\mathbf{A}}} C^{ + }$ we need to know $C^{ + }$. It is straightforward to drive Eq. (31) for $C^{ + }$ by combining Eqs. (1–4).

31 $$C^{ + } = C^{ - } - \frac{{\kappa_{e} \,}}{F}\nabla^{2} \phi$$

Then, from Eq. (28), $\nabla_{{\mathbf{A}}} C^{ + }$ can be determined and finally $\sigma$ is obtained as:

32 $$\sigma \left( {x,y,z,t} \right) = - \frac{{\kappa_{e} \sigma_{0} }}{{F\,\,\hat{r}\left( {h,W,L} \right)}}\left( {A_{1} \left( {x,y,z,t} \right) + \sum\limits_{i = 2}^{4} {A_{i} \left( {x,y,z} \right)} } \right),$$

where

33 $$A_{1} = \hat{K}\left( {r_{M} \left( h \right)\frac{{\partial U_{1} }}{\partial x} + r_{ew} \left( W \right)\frac{{\partial U_{1} }}{\partial y} + r_{el} \left( L \right)\frac{{\partial U_{1} }}{\partial z}} \right),$$

34 $$A_{2} = r_{M} \left( h \right)\left( {\frac{{\partial^{3} U_{2} }}{{\partial x^{3} }} + \frac{{\partial^{3} U_{2} }}{{\partial x\partial y^{2} }} + \frac{{\partial^{3} U_{2} }}{{\partial x\partial z^{2} }}} \right),$$

35 $$A_{3} = r_{ew} \left( W \right)\left( {\frac{{\partial^{3} U_{2} }}{{\partial y\partial x^{2} }} + \frac{{\partial^{3} U_{2} }}{{\partial y^{3} }} + \frac{{\partial^{3} U_{2} }}{{\partial y\partial z^{2} }}} \right),$$

36 $$A_{4} = r_{el} \left( L \right)\left( {\frac{{\partial^{3} U_{2} }}{{\partial z\partial x^{2} }} + \frac{{\partial^{3} U_{2} }}{{\partial z\partial y^{2} }} + \frac{{\partial^{3} U_{2} }}{{\partial z^{3} }}} \right).$$

$U_{1}$ and $U_{2}$ already were introduced in Eqs. (12) and (13).

### The bending moment

The global bending moment of a cantilevered IPMC beam, where the stimulus is applied at the clamped end of the beam (z = 0), is described by a vector like **M** as follows:

37 $${\mathbf{\rm M}} = \int\limits_{{c_{y} }} {\int\limits_{{c_{x} }} {{\mathbf{r}} \times \left( {\sigma_{zx} \,{\mathbf{i}} + \sigma_{zy} \,{\mathbf{j}} + \sigma_{zz} \,{\mathbf{k}}} \right)} } \,dx\,dy,$$

ans **r** is the position vector and is defined by ${\mathbf{r}} = x\,{\mathbf{i}} + y\,{\mathbf{j}}$. Equation (37) can be expressed in terms of:

38 $${\mathbf{\rm M}} = M_{zx} {\mathbf{i}} + M_{zy} {\mathbf{j}} + M_{zz} {\mathbf{k}},$$

39 $$M_{zx} = \int\limits_{{c_{y} }} {\int\limits_{{c_{x} }} {y\,\sigma_{zz} \,} } \,dx\,dy,$$

40 $$M_{zy} = \int\limits_{{c_{y} }} {\int\limits_{{c_{x} }} {x\,\sigma_{zz} } } \,dx\,dy,$$

41 $$M_{zz} = \int\limits_{{c_{y} }} {\int\limits_{{c_{x} }} {\left( {x\,\sigma_{zy} - y\,\sigma_{zx} } \right)\,} } dx\,dy.$$

**Figure 4:**
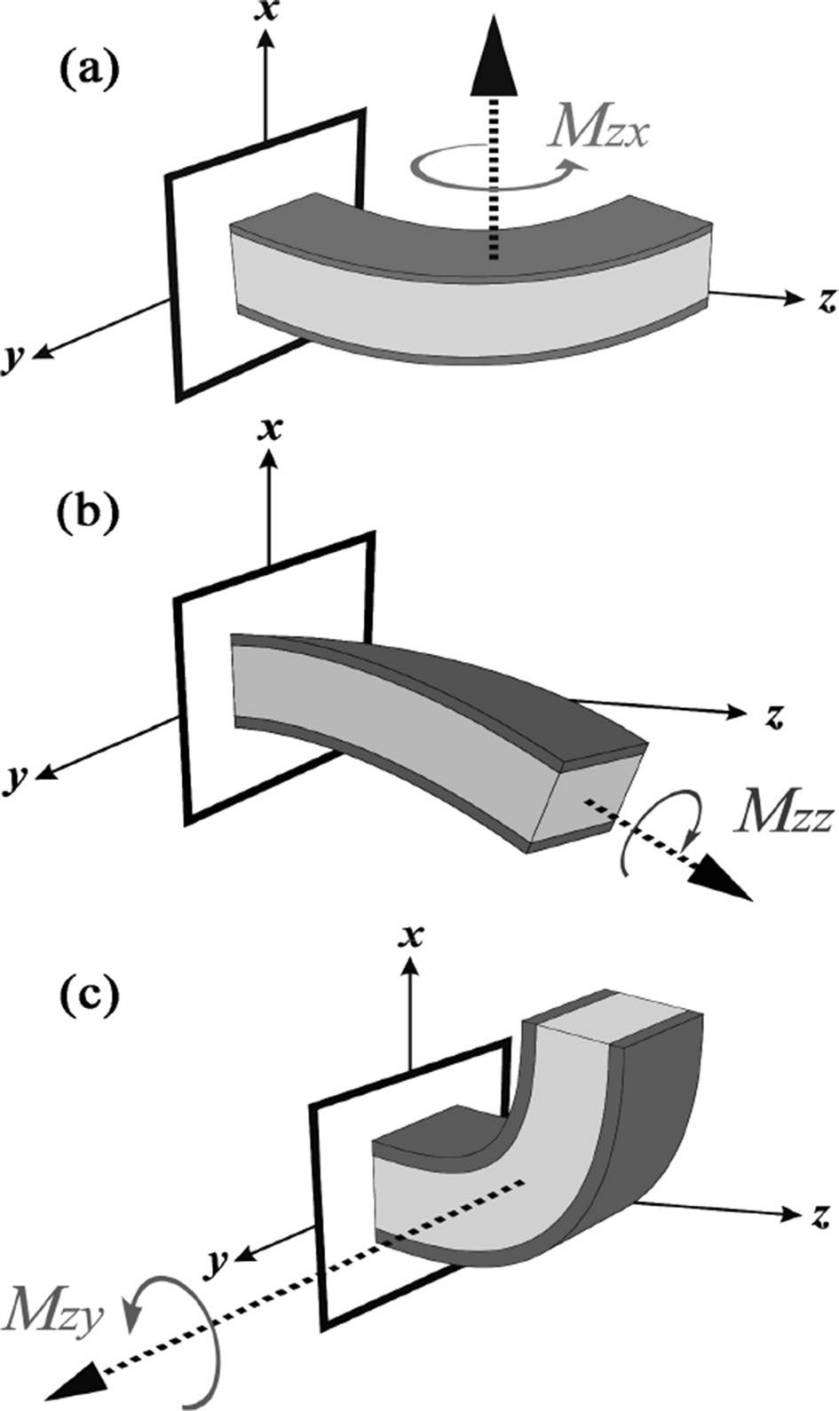
Three possible bending moments for an IPMC beam. (**a**) *M*_*zx*_ is exactly zero, (**b**) *M*_*zz*_ is almost zero, (**c**) *M*_*zy*_ dominates the IPMC bending in the direction of the *x*-axis.

Figure 4 shows our geometric definitions of an IPMC beam in the cantilevered configuration. As illustrated in Fig. 4, *M*_*zx*_ is exactly zero, *M*_*zz*_ is almost zero and only *M*_*zy*_ dominates the IPMC bending in the direction of the *x*-axis. Indeed, the only effective bending moment, *M*_*zy*_, can be expressed as:

42 $$M_{zy} \left( {z,t} \right) = \int_{0}^{W} {\int_{ - h}^{ + h} {x\,\sigma_{zz} \left( {x,y,z,t} \right)\,dxdy} } ,$$

$\sigma_{zz} \left( {x,y,z,t} \right)$ is the renamed version of $\sigma \left( {x,y,z,t} \right)$ defined in Eq. (32). Then, by substituting Eqs. (32–36) into Eq. (42) and solving integration terms, *M*_*zy*_ is determined as Eq. (43).

43 $$M_{zy} (z,t) = - \frac{{\kappa_{e} \sigma_{0} }}{{F\,\,\hat{r}\left( {h,W,L} \right)}}\left( {M_{1\,zy} (z,t) + M_{2zy} (z)} \right)$$

$M_{1\,zy} (z,t)$ and $M_{2\,zy} (z)$ are defined as Eqs. (44) and (45) respectively.

44 $$\begin{aligned} M_{1\,zy} (z,t) &= - \left( {\frac{{2\,\hat{K}\,\hat{\alpha }_{3} \left( t \right)\,r_{el} \left( L \right)\,}}{{\hat{\alpha }_{1} \left( t \right)^{2} \hat{\alpha }_{2} }}} \right) \hfill \\ &\quad \times \left( {1 - \hat{\alpha }_{1} \left( t \right)\,\coth \left( {\hat{\alpha }_{1} \left( t \right)\,h} \right)} \right)\left( {\tanh (\frac{{\hat{\alpha }_{2} W}}{2})} \right) \hfill \\ &\quad \times \left( {a_{L} \left( {V_{I} \left( t \right)} \right){\text{csch}} \left( {\hat{\alpha }_{3} \left( t \right)\,L} \right) - \coth \left( {\hat{\alpha }_{3} \left( t \right)\,L} \right)} \right) \hfill \\ &\quad \times \cosh \left( {\hat{\alpha }_{3} \left( t \right)\,z} \right)\,\,V_{I} \left( t \right), \hfill \\ \end{aligned}$$

45 $$M_{2zy} (z) = - \left( {\frac{{2h^{3} W\,b_{mnp} }}{3}} \right)\left( {\hat{\Upsilon }_{2} + \hat{\Upsilon }_{5} \,\frac{W}{2} + \hat{\Upsilon }_{6} \,W\,z} \right).$$

Here, "csch(.)" is the hyperbolic cosecant operation and $b_{mnp}$ is a constant coefficient that is related to the numbers of harmonics in calculating $U_{2}$ and $\hat{\Upsilon }_{2}$, also,$\hat{\Upsilon }_{5}$ and $\hat{\Upsilon }_{6}$ are constant coefficients that are produced through the combination of $\Upsilon_{1}$ to $\Upsilon_{8}$ and $\gamma_{1}$ to $\gamma_{3}$ and we should predict them in the model prediction stage. Consequently, *M*_*zy*_ can be rewrite as Eq. (46).

46 $$M_{zy} (z,t) = \tilde{\Upsilon }_{1} \left( t \right) + \tilde{\Upsilon }_{2} \left( t \right)z + \tilde{\Upsilon }_{3} \left( t \right)\cosh \left( {\hat{\alpha }_{3} \left( t \right)\,z} \right)$$

$\tilde{\Upsilon }_{1} \left( t \right)$ to $\tilde{\Upsilon }_{3} \left( t \right)$ are defined by Eqs. (47–49) respectively.

47 $$\tilde{\Upsilon }_{1} \left( t \right) = \left( {\frac{{\kappa_{e} \sigma_{0} }}{{F\,\,\hat{r}\left( {h,W,L} \right)}}t} \right)\left( {\frac{{2h^{3} W}}{3}} \right)\left( {\hat{\Upsilon }_{2} + \hat{\Upsilon }_{5} \,\frac{W}{2}} \right)\,b_{mnp}$$

48 $$\tilde{\Upsilon }_{2} \left( t \right) = \left( {\frac{{\kappa_{e} \sigma_{0} }}{{F\,\,\hat{r}\left( {h,W,L} \right)}}t} \right)\left( {\frac{{2h^{3} W}}{3}} \right)\left( {\hat{\Upsilon }_{6} \,W} \right)\,b_{mnp}$$

49 $$\begin{aligned} \tilde{\Upsilon }_{3} \left( t \right) &= \left( {\frac{{\kappa_{e} \sigma_{0} }}{{F\,\,\hat{r}\left( {h,W,L} \right)}}t} \right)\left( {\frac{{2\,\hat{K}\,\hat{\alpha }_{3} \left( t \right)\,r_{el} \left( L \right)\,}}{{\hat{\alpha }_{1} \left( t \right)^{2} \hat{\alpha }_{2} }}} \right) \hfill \\ &\quad \times \left( {1 - \hat{\alpha }_{1} \left( t \right)\,\coth \left( {\hat{\alpha }_{1} \left( t \right)\,h} \right)} \right)\left( {\tanh (\frac{{\hat{\alpha }_{2} W}}{2})} \right) \hfill \\ &\quad \times \left( {a_{L} \left( {V_{I} \left( t \right)} \right){\text{csch}} \left( {\hat{\alpha }_{3} \left( t \right)\,L} \right) - \coth \left( {\hat{\alpha }_{3} \left( t \right)\,L} \right)} \right)\,\,V_{I} \left( t \right) \hfill \\ \end{aligned}$$

### Calculation of the IPMC tip displacement in a large deformation situation

To calculate the IPMC tip displacement, first, we need to determine its shape function. Most of the previous works have modeled the mechanical vibration of the IPMC through linear Euler–Bernoulli (LEB) cantilever beam theory incorporating damping and accommodating suitable boundary conditions^16,30,32,33,42^. Based on LEB cantilever beam theory, the relation between the shape function of the IPMC,$\omega \left( {z,t} \right)$, and its bending moment, $M_{zy} \left( {z,t} \right)$, is defined as follows:

50 $$\frac{{\partial^{2} \omega \left( {z,t} \right)}}{{\partial z^{2} }} = \frac{{M_{zy} \left( {z,t} \right)}}{Y\,I},$$

*Y* is Young's modulus of the Nafion membrane, and *I* is the moment of inertia and calculated by the following equation:

51 $$I = \int_{0}^{L} {\int_{0}^{W} {\int_{ - h}^{ + h} {\left( {x^{2} + z^{2} } \right)\,dx\,dy\,dz} } } \Rightarrow I = \frac{2}{3}h^{3} WL.$$

However, LEB cantilever beam theory is valid just for infinitesimal strains which means this theory can only model small deformation (vibration) of a beam. Hence, we cannot use it for modeling large deformation situations of an IPMC beam.

Mathematically the curvature of a function like $\omega \left( {z,t} \right)$ can be calculated by $\kappa_{Math} \left( {z,t} \right)$ which is defined as follows:

52 $$\kappa_{Math} \left( {z,t} \right) = \frac{{\frac{{\partial^{2} \omega \left( {z,t} \right)}}{{\partial z^{2} }}}}{{\left( {1 + \left( {\frac{{\partial \omega \left( {z,t} \right)}}{\partial z}} \right)^{2} } \right)^{\frac{3}{2}} }}.$$

On the other hand, the curvature of a beam under an applied bending moment,$M_{zy} \left( {z,t} \right),$ is mechanically defined by:

53 $$\kappa_{Mech} \left( {z,t} \right) = \frac{{M_{zy} \left( {z,t} \right)}}{Y\,I}.$$

Consequently, our aim is to find a function like $\omega \left( {z,t} \right)$ that its curvature matches with the curvature of a cantilever beam (IPMC) with an applied bending moment $M_{zy} \left( {z,t} \right)$ to it. To find this function, $\kappa_{Math} \left( {z,t} \right)$ is set equal to $\kappa_{Mech} \left( {z,t} \right)$ and the resulted PDE is solved. Then, the following nonlinear PDE is obtained.

54 $$\begin{gathered} \,\,\,\,\,\,\,\,\,\,\,\,\,\,\,\,\,\,\,if\,\,\,\kappa_{Math} \left( {z,t} \right) = \kappa_{Mech} \left( {z,t} \right) \hfill \\ \Rightarrow \frac{{\partial^{2} \omega \left( {z,t} \right)}}{{\partial z^{2} }} - \left( {\frac{{M_{zy} \left( {z,t} \right)}}{Y\,I}} \right)\left( {1 + \left( {\frac{{\partial \omega \left( {z,t} \right)}}{\partial z}} \right)^{2} } \right)^{\frac{3}{2}} = 0 \hfill \\ \end{gathered}$$

#### Lemma 3

*In Eq. *(54),* if we ignore the square of the beam slope (i.e. term *$\left( {\frac{{\partial \omega \left( {z,t} \right)}}{\partial z}} \right)^{2}$*), we reach to the PDE resulted by LEB beam theory (Eq. **50**) for small deformations.*

*Equation* (54) *is a nonlinear PDE but it is solvable analytically. So*, $\omega \left( {z,t} \right)$ *will be obtained as follows*:

55 $$\omega \left( {z,t} \right) = \frac{{f\left( t \right)\,Ln\left( {P\left( {z,t} \right)} \right) + S(t)\,q\left( {z,t} \right)}}{{\left( {\frac{f\left( t \right)}{{g\left( t \right)}}} \right)Ln\left( {\frac{1}{{P\left( {0,t} \right)}}} \right) - S(t)\,q\left( {0,t} \right)}} + g\left( t \right)\,,$$

*where*

56 $$P\left( {z,t} \right) = \left| {\frac{{f\left( t \right)\tan \left( {\frac{{q\left( {z,t} \right)}}{2}} \right) + S(t) - Y\,I}}{{f\left( t \right)\tan \left( {\frac{{q\left( {z,t} \right)}}{2}} \right) - S(t) - Y\,I}}} \right|,$$

57 $$S(t) = \sqrt {\left( {YI - f\left( t \right)} \right)\left( {YI + f\left( t \right)} \right)} ,$$

58 $$q\left( {z,t} \right) = Arc\sin \left( {\frac{{m\left( {z,t} \right) + f\left( t \right)}}{Y\,I}} \right),$$

59 $$\begin{gathered} \,\,m\left( {z,t} \right) = \int {M_{yz} \left( {z,t} \right)} \,dz \hfill \\ \,\,\,\,\,\,\,\,\,\,\,\,\,\,\,\,\,\, = \tilde{\Upsilon }_{1} \left( t \right)z + \tilde{\Upsilon }_{2} \left( t \right)\frac{{z^{2} }}{2} + \tilde{\Upsilon }_{3} \left( t \right)\frac{{\sinh \left( {\hat{\alpha }_{3} \left( t \right)\,z} \right)}}{{\hat{\alpha }_{3} \left( t \right)}}, \hfill \\ \end{gathered}$$

60 $$f\left( t \right) = k_{f} \,e^{{ - \,\frac{t}{{\tau_{f} }}}} ,$$

61 $$g\left( t \right) = k_{g} \,e^{{ - \,\frac{t}{{\tau_{g} }}}} .$$

$k_{f}$,$k_{g}$,$\tau_{f}$ and $\tau_{g}$ *are constant coefficients and should be estimated in the model estimation stage*.

*The curve* $\omega \left( {z,t} \right)$ *describes the deflection of the beam at some position z. To find the tip displacement of the beam (IPMC) at each moment we name the IPMC tip displacement as *$\delta_{Tip} \left( t \right)$. *Some previously published papers like* ^32^, *have used* Eq. (62) *which is an approximation of IPMC tip displacement for very small deformations.*

62 $$\delta_{Tip} \left( t \right) = \omega \left( {L,t} \right)$$

In Eq. (62), *L is the length of the IPMC. To extend this approximation to large deformations which we follow in this paper, we rewrite* *Eq*. (62) *as Eq*. (63).

63 $$\delta_{Tip} \left( t \right) = \omega \left( {z_{L} \left( t \right),t} \right)$$

$z_{L} \left( t \right)$ *describes the dynamic coordinate of z component of the IPMC’s tip as follows*:

64 $$\int_{0}^{{z_{L} \left( t \right)}} {\sqrt {1 + \left( {\frac{{\partial \omega \left( {z,t} \right)}}{\partial z}} \right)^{2} } dz \, } = L.$$

Equation (64) *describes the arc length of the IPMC that is constant and equal to L*.

#### Lemma 4

*In Eq. (**64**) if we ignore the square of the curve slope (i.e. term *$\left( {\frac{{\partial \omega \left( {z,t} \right)}}{\partial z}} \right)^{2}$*), we reach to Eq. (**62**) that is an approximation of IPMC tip displacement for very small deformations.*

### Parameter estimation and model validation

**Figure 5:**
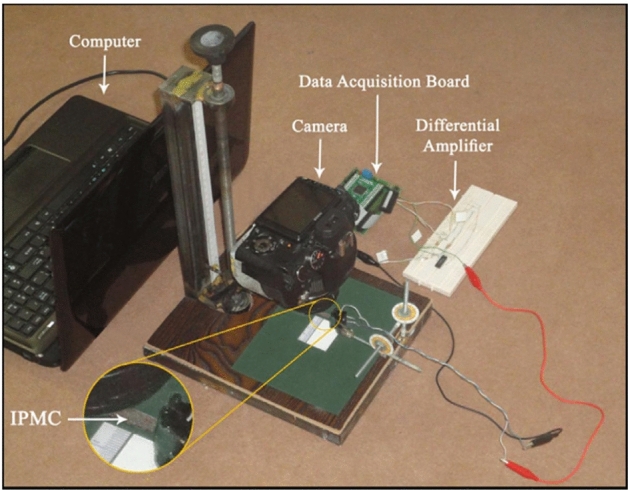
The photograph of hardware apparatus^9^.

In this section, we aim to validate our claims and find an accurate well-defined relationship between the input applied voltage ($V_{I} \left( t \right)$) and the output tip displacement of the IPMC ($\delta_{Tip} \left( t \right)$). Totally, to produce $V_{I} \left( t \right)$ and acquire $\delta_{Tip} \left( t \right)$, we used a set of hardware apparatus. As shown in this Fig. 5, the setup was composed of several components: a computer with MATLAB 2012b, a data acquisition board, a differential electronic amplifier, an accurate mechanical camera handler, a tin-plated copper clips, and a camera. To record the IPMC actuation data, the desired input signals ($V_{I} \left( t \right)$) were produced in MATLAB and written serial to send to the data acquisition board (DAQ board). The output voltage of one of the digital to analog converter (DAC) outputs of the DAQ was amplified and shifted by a designed differential amplifier and then applied to the IPMC using a clip. When the signal was applied to the IPMC, a video was taken of the IPMC’s actuation by a video camera, simultaneously. Then, by employing appropriate video and image processing techniques (we refer the reader to Appendix D), the required features were extracted from these video files ^9^. The sampling rate of the data acquisition was 29.7 S/s which was equal to the frame per second of the recorded video (29.7 FPS).

Table 1 summarizes specifications of the IPMC used in this research. In all physics-based models there are some specified physical parameters and some unknown parameters. The specified physical parameters were reported in the references and are listed in Table 2 but unknown parameters should be estimated in the parameter estimation stage.

**Table 1:** Specifications of the IPMC strip used in this research.

Specification	Description
Length × Width × Thickness	28 mm × 6 mm × 0.2 mm
Membrane material	Dupont™ Nafion 117
Electrode and cations	Platinum and Na +

**Table 2:** Specified physical parameters.

$F = 96458\,\left[ \frac{C}{mol} \right]$	$k_{e} = 1.34 \times 10^{ - 6} \,\left[ \frac{F}{m} \right]$
$Y = 5.71 \times 10^{8} \,\left[ {Pa} \right]$	$C^{ - } = 1200\,\left[ {\frac{mol}{{m^{3} }}} \right]$
$d = 1.03 \times 10^{ - 11} \,\left[ {\frac{{m^{2} }}{s}} \right]$	$R = 8.3143\,\left[ {\frac{J}{mol.K}} \right]$
$T = 293\,\left[ K \right]$	$L \times W \times h = 28 \times 6 \times 0.0889\,\left[ {mm^{3} } \right]$

For parameter estimation and validation of the model we used the GA toolbox of MATLAB software. Three different signals were applied as the input voltage to the IPMC and measured its tip displacement in response to each stimulus. Applied stimulus signals were chirp, Pseudo Random Binary Sequence (PRBS) and sinusoidal signals with a peak voltage of 2.3 V. Then, we estimated the unknown parameters of the proposed model using the half of each input–output data pairs (voltage as the input and the tip displacement of the IPMC in the *x*-direction as the output). For model validation the defined model was tested by the rest of the data pairs.

**Figure 6:**
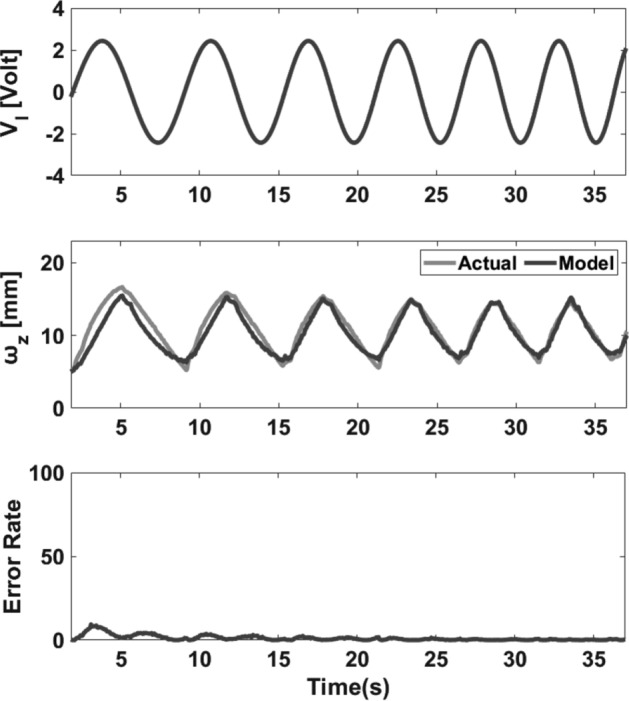
Model validation. Top: applied chirp voltage (with increasing frequency (up-Chirp)) to the IPMC (with a peak voltage of 2.3 V). Middle: the actual and estimated tip displacement of the IPMC in the *x-*direction. Bottom: the square error rate.

**Figure 7:**
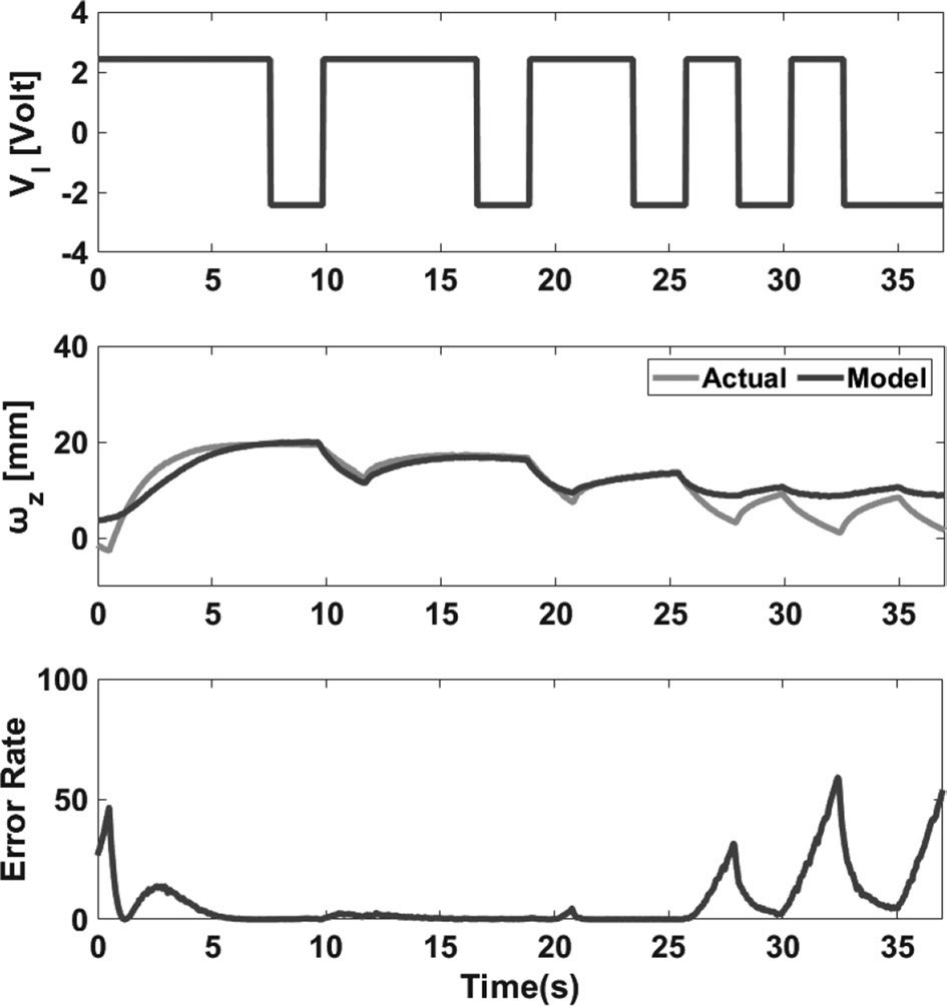
Model validation. Top: applied PRBS voltage to the IPMC (with a peak voltage of 2.3 V). Middle: the actual and estimated tip displacement of the IPMC in the *x-*direction. Bottom: the square error rate.

**Figure 8:**
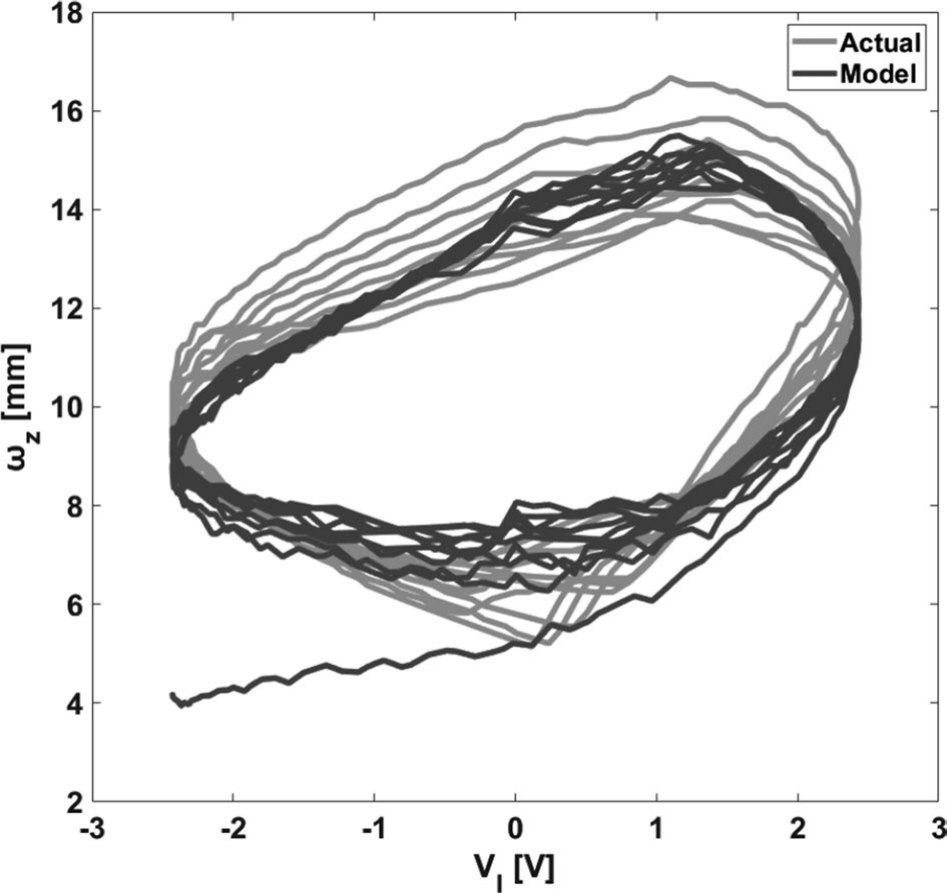
Displacement vs. voltage state-trajectory of chirp dataset.

There are a variety of classic and intelligent methods to estimate unknown parameters and we chose a genetic algorithm-based (GA) optimization method to find unknown parameters. The estimated parameters are reported in Table 3. Figures 6 and 7 illustrate the experimental tests of the model for Chirp and PRBS applied voltages. Moreover, the displacement vs. voltage state-trajectory for chirp dataset is shown in Fig. 8. Results indicate that the proposed model follows the actual output precisely. Moreover, to quantify the accuracy of the model by a numerical criterion, the normalized mean-square error (NMSE) was selected. The NMSE of the model in response to sinusoidal, PRBS, and chirp input signals are 0.07, 0.025 and 0.0047, respectively. Accordingly, the proposed model is accurate and can work properly in large deformation situations and practical applications. Furthermore, we compared the result of the proposed model with two other methods, Laguerre expansion method (LEM) and ANFIS-NARX paradigm^10^. The reason for choosing these two methods is that we used only the information of the input to predict the output in the proposed model which means the proposed method is a non-autoregressive model, hence, we should compare our model with other non-autoregressive methods. One of the best non-autoregressive models is Laguerre Expansion Technique (LET)^43^ that in Table 4 we compared the NMSE of the proposed model with LET. The mean NMSE of the proposed model is about 24 times smaller than the mean NMSE of LEM which means that, the proposed model is 24 times more accurate than LEM. As the second method, we compared the proposed model with ANFIS-NARX paradigm. ANFIS-NARX paradigm is an autoregressive method that is valid for IPMC large deformation situation^10^. As reported in Table 4, ANFIS–NARX paradigm is more accurate than the proposed model, but it doesn’t mean it is more appropriate because ANFIS–NARX is an autoregressive model which uses output feedback to achieve a precise prediction model. Using output data for an accurate modeling eliminates the causal relationship between the applied voltage and the IPMC bending. Moreover, in most of practical applications acquiring output feedback of the IPMC is not feasible. Hence, from the viewpoint of practical applications, a non-autoregressive model even with less accuracy is preferred to a more accurate autoregressive model.

**Table 3:** Estimated parameters using genetic algorithm.

$k_{f}$	0.5	$\hat{\Upsilon }_{2}$	0.04	$r_{el}$	0.11
$\tau_{f}$	44	$\hat{\Upsilon }_{5}$	0.03	$\mu$	2.4
$k_{g}$	4.5	$\hat{\Upsilon }_{6}$	5 × 10^–3^	$\sigma_{0}$	0.10
$\tau_{g}$	0.07	$r_{M}$	0.15		
$B_{mnp}$	0.003	$r_{ew}$	0.175

**Table 4:** NMSE of the proposed method in comparison with the LEM and ANFIS–NARX methods for PRBS training data set.

	Proposed model	LEM	ANFIS-NARX
SINE	0.07	0.911	2.38e−5
PRBS	0.025	0.828	6.39e−6
CHIRP	0.0047	0.634	2.42e−6
MEAN	**0.0332**	**0.790**	**1.08e−5**

## Conclusion

It this paper a fully analytical and physics-based 3D ion transport model was presented for large deformable IPMC actuators. This model exhibits a compact and explicit relationship between the input voltage and the output tip displacement of the IPMC actuator which is uniquely valid for large deformation situation. During the various stages of modeling, four Lemmas were presented which support that the proposed model passed a true way as well as the previous models for small deformations and moreover, is an explanation of them for large deformations. Furthermore, several experimental results were presented to demonstrate its applicability to arbitrary electrical inputs. The agreement between model predictions and experimental results also provides insight into the underlying actuating mechanisms of IPMC materials due to the physical based of the model.

## Supplementary Information

Supplementary Information 1.

Supplementary Video 1.

## Abbreviations

*h*: Half of the thickness of the Nafion membrane
*W*: Width of the IPMC beam
*L*: Length of the IPMC beam
*t*_*h*_: Thickness of the Pt electrode
***D***(**x**,**y**,**z**,**t**): Electric displacement
***E***(**x**,**y**,**z**,**t**): Electric field
$$\phi \left( {x,y,z,t} \right)$$: Electric potential
$\rho \left( {x,y,z,t} \right)$: Electric charge density
$\kappa_{e}$: Dielectric permittivity of Nafion
*F*: Faraday constant
*C*^+^(*x,y,z,t*): Cation concentration
$$C^{ - }$$: Anion concentration
***J***(**x**,**y**,**z**,**t**): Cation flux vector
*d*: Ionic diffusivity
*R*: Gas constant
*T*: Absolute temperature
*p*: Fluid pressure
**v**: Free solvent velocity field
$$\Delta V$$: Volumetric change
$k^{\prime}$: Hydraulic permeability coefficient
*V*_*I*_*(t)*: Input voltage applied to the IPMC
$${\upsigma }\left( {\text{x,y,z,t}} \right)$$: Mechanical stress
$\sigma_{0}$: Charge-stress coupling constant
**M**(*x,y,z,t*): Global bending moment
**r**: Position vector
*M*_*zy*_(*z,t*): Effective bending moment of the IPMC
*Y*: Young's modulus of Nafion
*I*: Moment of inertia
$\omega \left( {z,t} \right)$: Shape function of the IPMC
$\kappa_{Math} \left( {z,t} \right)$: Mathematical definition for the curvature of the shape function
$\kappa_{Mech} \left( {z,t} \right)$: Mechanical definition for the curvature of the shape function
$\delta_{Tip} \left( t \right)$: Tip displacement of the IPMC
