# Supplementary material for: A 3D analytical ion transport model for ionic polymer metal composite actuators in large bending deformations
Source: Sci Rep. 2021 Mar 19;11:6435. doi: 10.1038/s41598-021-85776-4 (PMC7979887; doi:10.1038/s41598-021-85776-4)
Supplement: Supplementary file 1 — Supplementary Information 1. [file 41598_2021_85776_MOESM1_ESM.doc]

[[1]](#footnote-2) 

**Appendix A. Electric Potential's PDE**

Darcy’s law is used to relate the free solvent velocity field to the pressure gradient and the electric field ,

$\mathbf{v}=k'\left(C^{-}F \mathbf{E}-\nabla p\right) ,$ (A-1)

In (A-1), $k'$ is the hydraulic permeability coefficient. Neglecting the convection term (the second term in the right side of Eq. 6) , i.e. assuming **v**=0, leads to

$\nabla p=C^{-}F \mathbf{E} .$ (A-2)

Combining and substituting Eqs. (1)-(4), and (A-2) into the original ion flux equation (Eq. 6), one can rewrite **J** as

$\mathbf{J}=-d\left(\frac{\kappa_{e}}{F}\nabla\left(\nabla\cdot\mathbf{E}\right)-\frac{\kappa_{e}}{RT}\left(1-C^{-}\Delta V\right)\nabla\cdot\mathbf{E}\cdot\mathbf{E}-\right. \left.\frac{FC^{-}}{RT}\left(1-C^{-}\Delta V\right)\mathbf{E}\right) .$ (A-3)

Next, divergence operator () is applied to both sides of Eq. (A-3). Based on Eqs. (1) and (3) and vector calculations, terms,$\nabla^{2}\rho$ and $\left(\nabla\rho\right)\cdot\mathbf{E}+\kappa_{e}^{-1} \rho^{2}$are substituted into $\nabla\cdot\mathbf{J}$ instead of $\nabla\cdot\mathbf{E}$, $\nabla\cdot\left(\nabla\rho\right)$ and $\nabla\cdot\left(\rho\cdot\mathbf{E}\right)$, respectively.

$\nabla.\mathbf{J}=-d\left(\underbrace{\frac{1}{F}\nabla\rho^{2}}_{term 1}-\underbrace{\tilde{K}\left(\left(\nabla\rho\right).\mathbf{E}+\frac{1}{\kappa_{e}}\rho^{2}\right)}_{term 2}-\underbrace{\frac{FC^{-}}{\kappa_{e}}\tilde{K}\rho}_{term 3}\right) ,$ (A-4)

$\tilde{K}$ is

$\tilde{K}=\frac{1}{RT}\left(1-C^{-}\Delta V\right) .$ (A-5)

In Eq. (A-4), term 2 is much smaller than term 1 and term 3 . Neglecting term 2 results in Eq. (A-6).

$\nabla\cdot\mathbf{J}=-d\left(\frac{1}{F}\nabla\rho^{2}-\frac{FC^{-}}{\kappa_{e}}\tilde{K}\rho\right)$ (A-6)

Using Eqs. (A-6), (5) and (4), one obtains the following PDE for the charge density *ρ* inside the membrane.

$\frac{\partial\rho}{\partial t}=d \nabla^{2}\rho-K\rho$ (A-7)

$K=F^{2}C^{-}d \kappa_{e}^{-1}\tilde{K}$ and defined as Eq.(A-8).

$K=\frac{F^{2}C^{-}d\left(1-C^{-}\Delta V\right)}{RT\kappa_{e}}$ (A-8)

From Eqs. (1) to (3), Eq. (A-9) is obtained as a relation between the electric potential and the electric charge density.

(A-9)

Finally, substituting (A-9) into (A-7) results in the following time-variant 3D PDE for the electric potential.

(A-10)

**Appendix B. Derivation of equations (11-13) and (17-19)**

We start from an assumption that the answer of Eq. (7) is a separable function which leads to

(B-1)

By substituting (B-1) into (7), one gets

(B-2) $\frac{1}{T\left(t\right)}\frac{\partial T\left(t\right)}{\partial t}=d \frac{\nabla^{2}\left(\nabla^{2}U\left(x,y,z\right)\right)}{\nabla^{2}U\left(x,y,z\right)}-K.$

Then, it can be divided into two following equations:

(B-3) $\frac{1}{T\left(t\right)}\frac{\partial T\left(t\right)}{\partial t}=-\lambda,$

(B-4) $d \frac{\nabla^{2}\left(\nabla^{2}U\right)}{\nabla^{2}U}-K=-\lambda.$

It is supposed that$\lambda$ is a positive constant. The general solution of (B-3) is as (B-5). That, $k_{1}$is a positive constant.

(B-5) $T\left(t\right)=k_{1}e^{-\lambda t}$

To solve (B-4), we define a new function, *G*, as follows:

(B-6) $\nabla^{2}U\left(x,y,z\right)=G\left(x,y,z\right).$

So, the substitution of (B-6) into (B-4) results in

(B-7) $\nabla^{2}U=\frac{1}{\hat{K}}\nabla^{2}G ,$

(B-8) $\hat{K}=\frac{K-\lambda}{d}.$

The extended form of (B-7) is

|  | (B-9) |
| --- | --- |

(B-10) $\text{Indeed} \gamma_{1}+\gamma_{2}+\gamma_{3}=0 ,$

(B-11) $\frac{\partial^{2}U}{\partial x^{2}}-\frac{1}{\hat{K}}\frac{\partial^{2}G}{\partial x^{2}}=\gamma_{1} ,$

(B-12) $\frac{\partial^{2}U}{\partial y^{2}}-\frac{1}{\hat{K}}\frac{\partial^{2}G}{\partial y^{2}}=\gamma_{2} ,$

(B-13) $\frac{\partial^{2}U}{\partial z^{2}}-\frac{1}{\hat{K}}\frac{\partial^{2}G}{\partial z^{2}}=\gamma_{3} .$

Consequently, we get following solutions for (B-11) to (B-13).

(B-14) $U\left(x,y,z\right)=\frac{1}{\hat{K}}G\left(x,y,z\right)+\psi_{1}\left(x,y,z\right)$

(B-15) $U\left(x,y,z\right)=\frac{1}{\hat{K}}G\left(x,y,z\right)+\psi_{2}\left(x,y,z\right)$

(B-16) $U\left(x,y,z\right)=\frac{1}{\hat{K}}G\left(x,y,z\right)+\psi_{3}\left(x,y,z\right)$

(B-17) $\text{And} \psi_{1}\left(x,y,z\right)=\frac{\gamma_{1}}{2}x^{2}+\xi_{11}\left(y,z\right)x+ \xi_{12}\left(y,z\right) ,$

(B-18) $\psi_{2}\left(x,y,z\right)=\frac{\gamma_{2}}{2}y^{2}+\xi_{21}\left(x,z\right)y+ \xi_{22}\left(x,z\right) ,$

(B-19) $\psi_{3}\left(x,y,z\right)=\frac{\gamma_{3}}{2}z^{2}+\xi_{31}\left(x,y\right)z+ \xi_{32}\left(x,y\right) .$

$\xi_{ij}$are arbitrary functions. In order to present a unified solution for *U*(*x, y, z*) and also keeping (B-10) condition, we use union operator instead of (B-14) to (B-16) as follows:

(B-20) $\begin{matrix} U\left(x,y,z\right)=\left(\frac{1}{\hat{K}}G\left(x,y,z\right)+\psi_{1}\left(x,y,z\right)\right) \\ \bigcup\left(\frac{1}{\hat{K}}G\left(x,y,z\right)+\psi_{2}\left(x,y,z\right)\right) \\ \bigcup\left(\frac{1}{\hat{K}}G\left(x,y,z\right)+\psi_{3}\left(x,y,z\right)\right), \\ \Rightarrow U\left(x,y,z\right)=\frac{1}{\hat{K}}G\left(x,y,z\right)+\Psi\left(x,y,z\right). \end{matrix}$

and $\Psi\left(x,y,z\right)$is defined as

(B-21) $\Psi\left(x,y,z\right)=\psi_{1}\left(x,y,z\right)+\psi_{2}\left(x,y,z\right)+\psi_{3}\left(x,y,z\right).$

So, by combining (B-6) and (B-20), the following inhomogeneous Poisson PDE is obtained.

(B-22) $\nabla^{2}U\left(x,y,z\right)=\hat{K} U\left(x,y,z\right)-\hat{K} \Psi\left(x,y,z\right)$

Now, by applying the superposition principle, *U* (*x, y, z*) can be expressed as the summation of *U*_1_ (*x, y, z*) and *U*_2_ (*x, y, z*) in (B-23). Actually, *U*_1_ (*x, y, z*) is the zero-input response of (B-22) (where ψ (*x, y, z*) is zero) under excitation of the initial conditions and *U*_2_ (*x, y, z*) is the zero-state response under excitation of the external input.

(B-23) $U\left(x,y,z\right)= U_{1}\left(x,y,z\right)+U_{2}\left(x,y,z\right)$

Finally, the substitution of (B-23) and (B-5) into (B-1) results to (B-24) which means that Eq. (11) is obtained.

(B-24)

The governing PDEs for *U*_1_ (*x, y, z*) and *U*_2_ (*x, y, z*) are two other Poisson PDEs, (B-25) and (B-26), with boundary conditions as (B-27) and (B-28), respectively.

| $\nabla^{2}U_{1}\left(x,y,z\right)=\hat{K} U_{1}\left(x,y,z\right)$ | (B-25) |
| --- | --- |
| $\nabla^{2}U_{2}\left(x,y,z\right)=\hat{K} U_{2}\left(x,y,z\right)-\hat{K} \Psi\left(x,y,z\right)$ | (B-26) |
| $\left\{\begin{matrix} U_{1}\left(+h,0,0\right)=\frac{V_{I}\left(t\right)}{2}, U_{1}\left(-h,0,0\right)=-\frac{V_{I}\left(t\right)}{2} \\ U_{1}\left(+h,0,L\right)=a_{L}\left(V_{I}\left(t\right)\right)\frac{V_{I}\left(t\right)}{2},U_{1}\left(-h,0,L\right)=-a_{L}\left(V_{I}\left(t\right)\right)\frac{V_{I}\left(t\right)}{2} \\ U_{1}\left(+h,W,0\right)=\frac{V_{I}\left(t\right)}{2}, U_{1}\left(-h,W,0\right)=-\frac{V_{I}\left(t\right)}{2} \\ U_{1}\left(+h,W,L\right)=a_{L}\left(V_{I}\left(t\right)\right)\frac{V_{I}\left(t\right)}{2}, U_{1}\left(-h,W,L\right)=-a_{L}\left(V_{I}\left(t\right)\right)\frac{V_{I}\left(t\right)}{2} \end{matrix}\right.$ | |
| (B-27) | |
| $\left\{\begin{matrix} U_{2}\left(+h,0,0\right)=0, U_{2}\left(-h,0,0\right)=0 \\ U_{2}\left(+h,0,L\right)=0, U_{2}\left(-h,0,L\right)=0 \\ U_{2}\left(+h,W,0\right)=0, U_{2}\left(-h,W,0\right)=0 \\ U_{2}\left(+h,W,L\right)=0, U_{2}\left(-h,W,L\right)=0 \end{matrix}\right.$ | (B-28) |

To solve (B-25), we suppose that

(B-29) $U_{1}\left(x,y,z\right)= X\left(x\right) Y\left(y\right) Z\left(z\right).$

Therefore, the substitution of (B-29) into (B-25) results in

|  | (B-30) |
| --- | --- |

that (as the same as Eq.(17) in the main text of the paper)

(B-31) $\hat{\alpha}_{1}^{2}+\hat{\alpha}_{2}^{2}+\hat{\alpha}_{3}^{2}=\hat{K},$

and we get

(B-32) $X\left(x\right)=\frac{V_{I}\left(t\right)}{2}\left(\frac{Sinh\left(\hat{\alpha}_{1} x\right)}{Sinh\left(\hat{\alpha}_{1} h\right)}\right),$

(B-33) $Y\left(y\right)=\left(\frac{1-cosh\left(\hat{\alpha}_{2}W\right)}{Sinh\left(\hat{\alpha}_{2}W\right)}\right)Sinh\left(\hat{\alpha}_{2}y\right)+cosh\left(\hat{\alpha}_{2}y\right),$

| $Z\left(z\right)=\left(\frac{a_{L}\left(V_{I}\left(t\right)\right)-cosh\left(\hat{\alpha}_{3}\left(t\right)L\right)}{sinh\left(\hat{\alpha}_{3}\left(t\right)L\right)}\right)sinh\left(\hat{\alpha}_{3}\left(t\right)z\right) +cosh\left(\hat{\alpha}_{3}\left(t\right)z\right).$ | (B-34) |
| --- | --- |

Finally, by combining (B-32)-(B-34) with (B-29), the following equation is obtained, which is the same as Eq. (12) in the main text of the paper.

| $\begin{matrix} U_{1}\left(x,y,z,t\right)=\left\{ \frac{V_{I}\left(t\right)}{2}\left(\frac{Sinh\left(\hat{\alpha}_{1} x\right)}{Sinh\left(\hat{\alpha}_{1}h\right)}\right)\right. \text{(A-35)} \\ \times\left(\left(\frac{1-cosh\left(\hat{\alpha}_{2}W\right)}{Sinh\left(\hat{\alpha}_{2}W\right)}\right)Sinh\left(\hat{\alpha}_{2}y\right)+cosh\left(\hat{\alpha}_{2}y\right)\right) \\ \times\left(\frac{a_{L}\left(V_{I}\left(t\right)\right)-cosh\left(\hat{\alpha}_{3}\left(t\right)L\right)}{sinh\left(\hat{\alpha}_{3}L\right)}\right)\times\left. \left(sinh\left(\hat{\alpha}_{3}z\right)+cosh\left(\hat{\alpha}_{3}z\right)\right) \right\} \end{matrix}$ |
| --- |

In the next step, to solve (B-26), we utilized the expansion of Fourier orthogonal functions and we assumed that the solution of (B-26) is defined as Eq. (13) in the main text of the paper. By applying it to (B-26) and doing some mathematical computation, the following equation is obtained.

(B-36) $\hat{K} \Psi\left(x,y,z\right)= \sum_{m=0}^{\infty} \sum_{n=0}^{\infty} \sum_{p=0}^{\infty} A_{mnp} \sin\frac{\left(m+1\right)\pi}{2h}x \sin\frac{\left(n+1\right)\pi}{W}y \sin\frac{\left(p+1\right)\pi}{L}z$

$A_{mnp}= a_{mnp}\left(\left(\frac{\left(m+1\right)\pi}{2h}\right)^{2}+\left(\frac{\left(n+1\right)\pi}{W}\right)^{2}+\left(\frac{\left(p+1\right)\pi}{L}\right)^{2}+\hat{K}\right)$ (B-37)

Based on basic principles of Fourier series, $A_{mnp}$is defined as

(B-38) $A_{mnp}=\frac{8\hat{K}}{2hWL}\int_{-h}^{+h} \int_{0}^{W} \int_{0}^{L} \tilde{\Psi}\left(x,y,z\right) dx dy dz.$

Where (as the same as Eq.(19) in the main text of the paper)

$\begin{matrix} \tilde{\Psi}\left(x,y,z\right)=\Psi\left(x,y,z\right) \\ \times\left(\sin\frac{\left(m+1\right)\pi}{2h}x \sin\frac{\left(n+1\right)\pi}{W}y \sin\frac{\left(p+1\right)\pi}{L}z\right). \end{matrix}$ (B-39)

Consequently,$a_{mnp}$is obtained as (B-40), which is the same as Eq. (18) in the main text of the paper.

| $a_{mnp}= \frac{\frac{8\hat{K}}{2hWL}\int_{-h}^{+h} \int_{0}^{W} \int_{0}^{L} \tilde{\Psi}\left(x,y,z\right) dx dy dz}{\left(\left(\frac{\left(m+1\right)\pi}{2h}\right)^{2}+\left(\frac{\left(n+1\right)\pi}{W}\right)^{2}+\left(\frac{\left(p+1\right)\pi}{L}\right)^{2}+\hat{K}\right)}$ | (B-40) |
| --- | --- |

**Appendix C. Determining** $\hat{\alpha}_{1}\left(t\right)$, $\hat{\alpha}_{2}$**and**$\hat{\alpha}_{3}\left(t\right)$ **in equations (14-16)**

Let' consider Fig. C-1 for the geometric definition of an IPMC beam with some more details.

Fig. C-1. The geometric definition of an IPMC beam with some more details.

The resistance of the electrodes defines as follows:

(C-1) $R=\rho_{s}\frac{L}{A}=\frac{\rho_{s}L}{t_{h}\times W}=\left(\frac{\rho_{s}}{t_{h}}\right)\frac{L}{W}\Rightarrow R=R_{s}\frac{L}{W},$

$\rho_{s}$,$A$, $t_{h}$and $R_{s}$are the specific electrical resistance, the cross-sectional area, the electrode thickness, and the sheet resistance of electrodes, respectively. As shown in Fig. C-1, $y=y_{a}$is a line in the direction of *z*-axis. In accordance with Ohm's law, the electric current passes through this path on the surface of the electrode is

(C-2)

Where is the electric potential.

By considering (C-1), ∆*R* is obtained as

(C-3) $\Delta R=R_{s}\frac{\Delta L}{W}.$

Now, by substituting (C-3) into (C-2), one gets

(C-4)

The right side of (C-4) is equal to

(C-5)

The left side is also equal to the voltage that is applied to this section of the surface ($V_{s}^{+} \left(z,t\right)$) divided by *W*. So, one gets to

(C-6)

As explained in section II-B in the main text of the paper, the applied voltage on the surface of the IPMC's electrode is $\frac{V_{I}\left(t\right)}{2}$and $a_{L}\left(V_{I}\left(t\right)\right)\frac{V_{I}\left(t\right)}{2}$ for the clamp part and the IPMC's tip, respectively. We suppose that $V_{s}^{+} \left(z,t\right)$can be any continues and smooth function that preserves these two conditions. A proper physics matched function for $V_{s}^{+} \left(z,t\right)$is defined as follows:

(C-7) $V_{s}^{+} \left(z,t\right)=\frac{V_{I}\left(t\right)}{2} \exp(\left(\frac{Ln \mathrm{a}_{L}\left(V_{I}\left(t\right)\right)}{L}\right)z) .$

Then, one can get (C-8) by inserting (C-7) and (B-24) in (C-6) and using (B-35) and Eq. (13) in the main text of the paper.

|  | (C-8) |
| --- | --- |

By putting value of z= y_a_=0 in (C-8), the following nonlinear equation is obtained for $\hat{\alpha}_{3}\left(t\right).$

(C-9) $\hat{\alpha}_{3}\left(\frac{a_{L}\left(V_{I}\left(t\right)\right)-cosh\left(\hat{\alpha}_{3}L\right)}{sinh\left(\hat{\alpha}_{3}L\right)}\right)=\frac{1}{W}-\frac{\partial U_{2}\left(+h,0,0\right)}{\partial z}$

The solution of Eq. (C-9) is

(C-10) $\hat{\alpha}_{3}\left(t\right)=\sqrt{\frac{2\theta}{L}Ln\left(\frac{4}{a_{L}\left(V_{I}\left(t\right)\right)L\theta}\right)}.$

$\theta$ is $1/W$. In a similar way, by putting value of z=0 and in (C-8), the following nonlinear equation is obtained for $\hat{\alpha}_{2}.$

(C-11) $\frac{1-cosh\left(\hat{\alpha}_{2}W\right)}{Sinh\left(\hat{\alpha}_{2}W\right)}=\left(\frac{\frac{1}{W}-\frac{\partial U_{2}\left(+h,y_{a},0\right)}{\partial z}}{\frac{1}{W}-\frac{\partial U_{2}\left(+h,0,0\right)}{\partial z}}\right)-\sqrt{2}$

The solution of Eq. (C-11) is

(C-12) $\hat{\alpha}_{2}=\frac{\mu}{W}Ln\left(1-\frac{1}{\mu^{2}}\right).$

And, *µ* is a constant coefficient.

Consequently, by substituting (C-10) and (C-12) into (B-31), $\hat{\alpha}_{1}\left(t\right)$is obtained as follows:

(C-13) $\hat{\alpha}_{1}\left(t\right)=\sqrt{\hat{K}-\left\{\frac{\mu^{2}}{W^{2}}\left(Ln\left(1-\frac{1}{\mu^{2}}\right)\right)^{2}+\frac{2\theta}{L}Ln\left(\frac{4}{a_{L}\left(V_{I}\left(t\right)\right)L\theta}\right)\right\}}.$

**Appendix D. The proposed image processing algorithm**

The proposed image processing algorithm was developed for a single frame of a whole video, and this method can be used on all frames of the video. The proposed method is represented in Fig D-1. The frame must be cropped in a specific boundary that is pre-adjusted, and then clarity of IPMC's shape will be enhanced by adjusting the brightness. The next step is converting the frame to a binary image, which will be useful for extracting the IPMC from the image's background. To find the tip of the IPMC, it is needed to decrease the size of the IPMC and take out a single-pixel line from it, and for this purpose, several morphological transforms were used on the binary image to obtain the single-pixel line. By using the size of the IPMC and the distance between the Camera and the plane in which the IPMC was held, the image's size would be changed to neglect the perspective effect. Then by using the 8-pixel neighborhood of each pixel, the tip of the IPMC will be fined. The coordination of the tip would be calculated concerning the clamp point in which IPMC was clamped. This coordination stored in IM_x_(t) and IM_y_(t). By applying this algorithm to all the frames, the coordination of IPMC's tip can be extracted for the entire video. These two vectors are more efficient to store and use than the whole video and help for future processes.


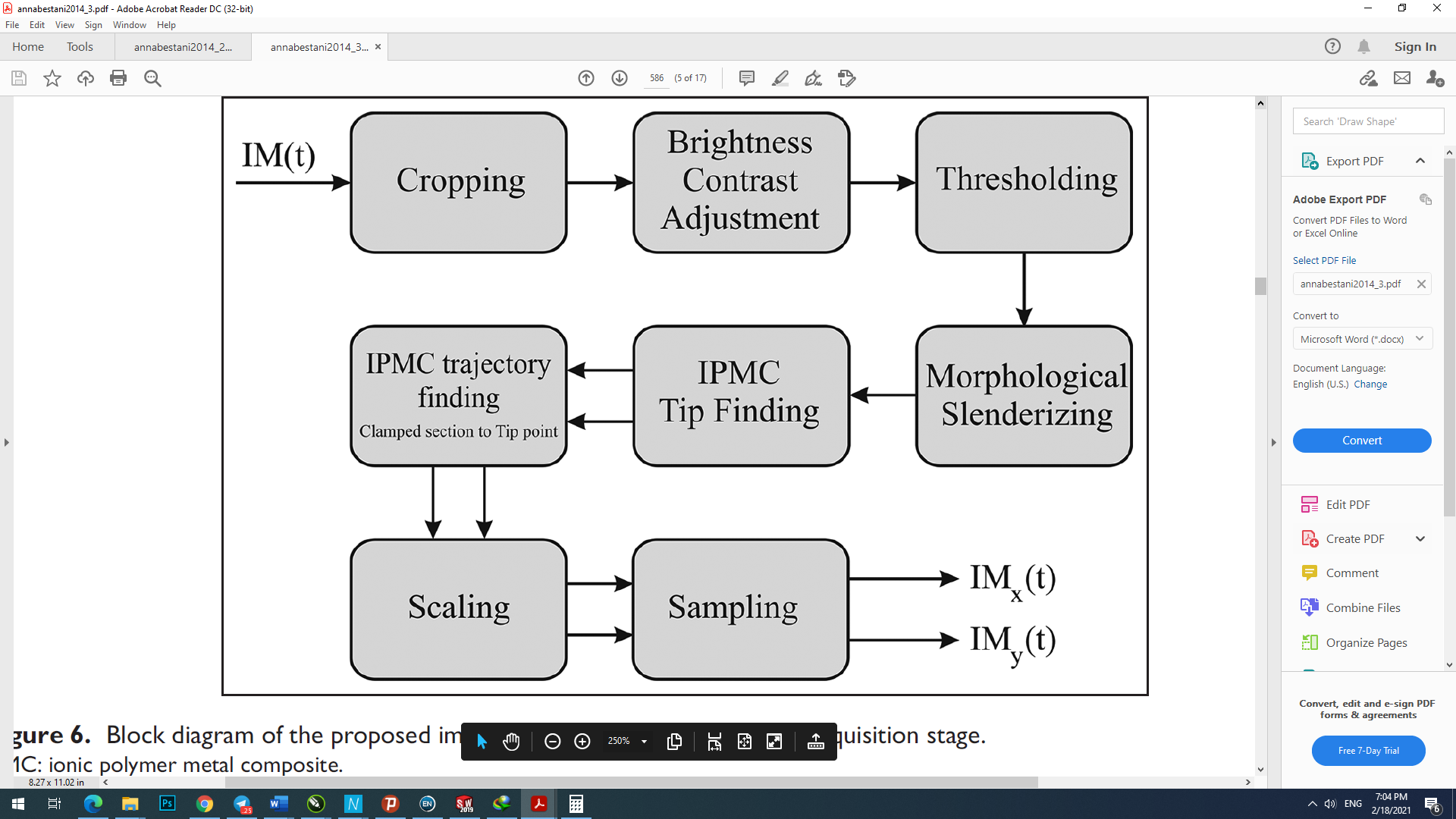


Fig. D-1. Block diagram of the proposed image processing algorithm.

1.  [↑](#footnote-ref-2)
